# Supplementary material for: Genome-Wide Analyses Reveal Genetic Convergence of Prolificacy between Goats and Sheep
Source: Genes (Basel). 2021 Mar 26;12(4):480. doi: 10.3390/genes12040480 (PMC8065816; doi:10.3390/genes12040480)
Supplement: Supplementary file 1 [file genes-12-00480-s001.zip › Additional Figure S1-S2.pdf]

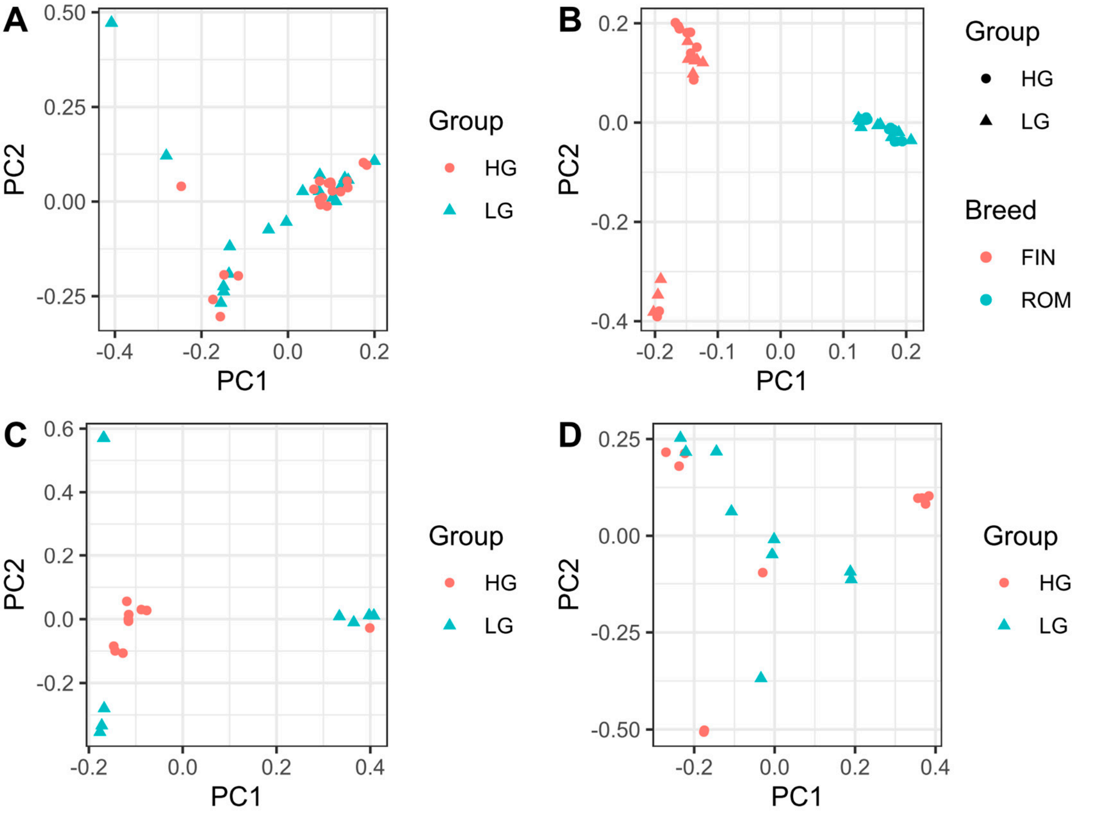


**Figure S1.** Principal component analysis of goats and sheep. (**A**) Yunshang black goats. (**B**) Finnsheep (FIN) and Romanov (ROM) sheep. (**C**) Finnsheep. (**D**) Romanov sheep. HG: high-yield group; LG: low-yield group.

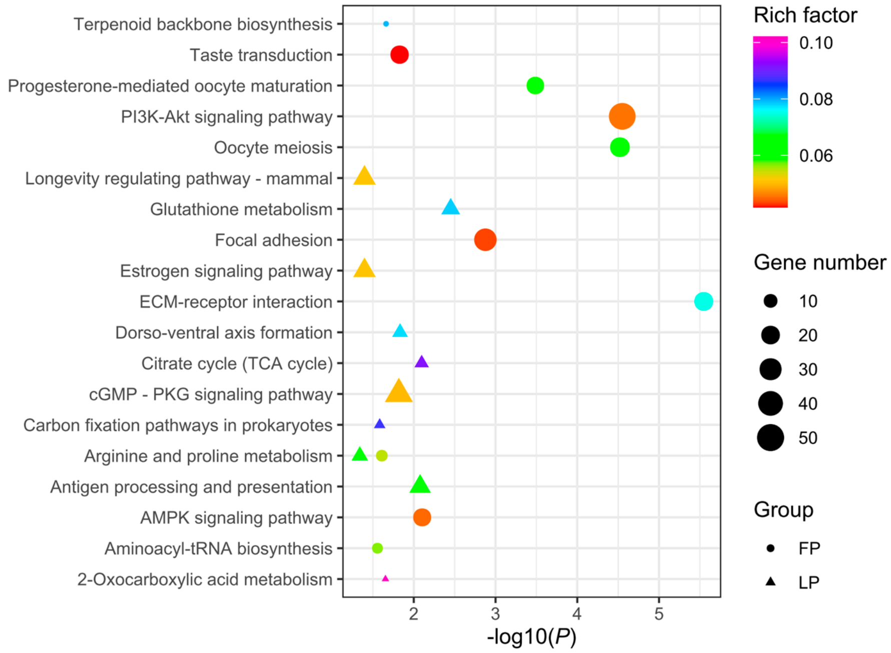


**Figure S2.** Top 10 KEGG terms of differentially expressed genes from luteal phase (LP) and follicular phase (FP) in Yunshang black goats.
